# Associations Between BioFire FilmArray Gastrointestinal Panel Results and Clinical Outcomes in Infectious Gastroenteritis

**DOI:** 10.3390/diagnostics15232947

**Published:** 2025-11-21

**Authors:** Myeong Joo Lee, Ju Yeong Lee, Suhng Wook Kim

**Affiliations:** Department of Health and Safety Convergence Science, Graduate School, Korea University, Seoul 02841, Republic of Korea; mjlee3048@cauhs.or.kr (M.J.L.); juyeong7@korea.ac.kr (J.Y.L.)

**Keywords:** infectious gastroenteritis (IGE), BioFire FilmArray gastrointestinal panel, Stool culture, antibiotic stewardship, hospitalization duration, rapid pathogen detection, infection control, Republic of Korea

## Abstract

**Background/Objectives**: Infectious gastroenteritis (IGE) is a major global health concern due to its high morbidity and healthcare burden. The BioFire FilmArray Gastrointestinal Panel (FA-GIP) enables rapid multiplex detection of enteric pathogens, offering faster results than conventional stool culture. This study aimed to evaluate the associations between FA-GIP result status and clinical outcomes in patients with suspected IGE. **Methods**: A retrospective analysis was conducted at Chung-Ang University Hospital (Seoul, Republic of Korea) from July 2023 to April 2024. Patients were stratified into FA-GIP-positive and FA-GIP-negative groups, and clinical parameters were compared. The diagnostic performance of FA-GIP was also assessed relative to stool culture. **Results**: No significant differences were observed in the demographic variables. However, the FA-GIP-positive group demonstrated significantly shorter hospital stays and time to discharge, and fewer antibiotic days, compared with the FA-GIP-negative group. Moreover, differences were observed in antibiotic modification or discontinuation rates. FA-GIP markedly shortened diagnostic turnaround time compared with culture (median 1.4 h vs. 72.3 h). **Conclusions**: These findings suggest that FA-GIP results are associated with clinically meaningful differences in patient management and antibiotic use. However, given the retrospective design, the relationship between FA-GIP testing and clinical decision-making should be interpreted as an association rather causative. Therefore, prospective studies are warranted to confirm the direct impact of FA-GIP-guided interventions on antibiotic stewardship and patient outcomes.

## 1. Introduction

Infectious gastroenteritis (IGE) remains a significant global public health concern, associated with high morbidity and mortality rates [1]. It is estimated that IGE is responsible for the death of approximately 2195 children each day [2]. As a leading cause of pediatric morbidity, IGE often necessitates medical consultation and hospitalization, imposing a substantial financial burden on healthcare systems [3,4]. The causative pathogens of IGE affect the gastrointestinal tract through diverse pathophysiological mechanisms. Given the complexity of these etiologies, timely identification of the causative pathogens is essential for their appropriate management [5]. Rapid detection supports the selection of effective treatment strategies and enables prompt intervention to prevent disease transmission.

For decades, conventional stool culture has been considered the gold standard for diagnosing bacterial gastroenteritis [6]. However, this method has significant limitations. For example, some bacteria do not readily grow in conventional culture media [7]. Additionally, stool culture is time-consuming, and its diagnostic accuracy may be compromised by factors such as inappropriate sample collection, particularly if samples are obtained after the initiation of antibiotic therapy. Other conventional diagnostic techniques, including microscopy, immunoassays, and real-time polymerase chain reaction (PCR), are labor-intensive and typically target a limited number of pathogens. When the causative pathogen remains unidentified and the clinical presentation is nonspecific, empirical therapy is often initiated based on clinical judgment [8].

Furthermore, antimicrobial prescribing practices among physicians may lack consistency. Increased use of inappropriate antibiotics for empirical therapy and reliance on broad-spectrum antibiotics for definitive treatment contribute to the emergence of multidrug-resistant organisms, perpetuating a vicious cycle [9]. Advancements in diagnostic technologies that ensure pathogen-targeted therapy and minimize complications are therefore critical for improving patient outcomes [10].

Recent advancements in multiplex molecular diagnostics have enabled rapid and simultaneous detection of multiple pathogens, representing a significant improvement in microbiological diagnostics [11]. One such platform is the FilmArray Gastrointestinal (GI) Panel (FA-GIP) (BioFire Diagnostics, Inc., Salt Lake City, UT, USA). FA-GIP is a molecular assay capable of detecting 22 bacterial, viral, and parasitic pathogens associated with IGE within a single platform. The assay requires a small sample volume (200 µL) and provides results within approximately 1 h. Compared with conventional methods, FA-GIP has demonstrated superior sensitivity and specificity, offering rapid and accurate pathogen identification [12,13].

Although several studies have evaluated the FA-GIP, the objective of this study was to investigate the associations between FA-GIP test results and clinical outcomes in patients with suspected IGE. We compared demographic and clinical parameters, including hospital stay, time to discharge, and patterns of antibiotic use, between FA-GIP-positive and FA-GIP-negative groups. In addition, the diagnostic performance of FA-GIP was assessed relative to conventional stool culture.

## 2. Materials and Methods

### 2.1. Research Design

This study retrospectively analyzed the medical records of 161 patients (aged 0–98 years) hospitalized with suspected infectious gastroenteritis (IGE) at the Chung-Ang University Hospital (CAUH) in Seoul, Republic of Korea, (July 2023–April 2024). Stool samples, collected as part of routine clinical care, were tested using the FA-GIP upon the request of infectious disease specialists. Cases with positive results solely for parasitic or viral pathogens were excluded, as this study focused on bacterial infections.

Clinical and demographic data, including patient age, sex, length of hospital stay (LOS), and antibiotic usage, were extracted from the medical records to assess clinical outcomes. All data were anonymized prior to analysis.

### 2.2. BioFire FilmArray

The FA-GIP is designed to detect 13 bacterial, 5 viral, and 4 parasitic pathogens. The analysis was performed using the FA-GIP according to the manufacturer’s instructions, and the results were reported to the attending clinicians. The FA-GIP system is designed to detect the following pathogens: bacteria, including *Campylobacter* spp. (*C. jejuni*, *C. coli*, and *C. upsaliensis*), *C. difficile* (toxin A/B), *Plesiomonas shigelloides, Salmonella* spp., *Yersinia enterocolitica*, *Vibrio* spp. (*V. parahaemolyticus*, *V. vulnificus*, and *V. cholerae*), *Vibrio cholerae*, as well as diarrheagenic *E. coli*/*Shigella*, including Enteroaggregative *E. coli* (EAEC), Enteropathogenic *E. coli* (EPEC), Enterotoxigenic *E. coli* (ETEC) *lt*/*st*, Shiga-like toxin-producing *E. coli* (STEC) *stx1*/*stx2*, *E. coli* O157, and *Shigella*/Enteroinvasive *E. coli* (EIEC), viruses, including Adenovirus F40/41, Astrovirus, Norovirus GI/GII, Rotavirus A, Sapovirus (I, II, IV, and V), and parasites, including *Cryptosporidium*, *Cyclospora cayetanensis*, *Entamoeba histolytica*, and *Giardia lamblia*.

### 2.3. Stool Culture

In our clinical microbiology laboratory, routine stool culture diagnostics involved inoculating stool samples onto blood agar (BAP), MacConkey agar (MAC), and selenite broth (SF broth). After 24 h of incubation, the SF broth was sub-cultured onto *Salmonella*–*Shigella* (SS) agar. For the detection of *Clostridioides difficile*, samples were inoculated onto ChromID *C. difficile* agar. *Campylobacter* spp. detection was performed by inoculating stool samples onto *Campylobacter*-selective agar and incubating them under microaerophilic conditions at 42 °C. After 48 h of incubation, bacterial colonies were analyzed, and suspected pathogenic colonies were identified using the VITEK MS system (bioMérieux, Marcy-l’Étoile, France) with Matrix-Assisted Laser Desorption Ionization–Time of Flight (MALDI-TOF) mass spectrometry. Additionally, *Salmonella* spp. were identified through antigen–antibody reaction testing using the Rbt-Ab™ *Salmonella* Agglutination Test-Poly (Joongkyeom Co., Ltd., Goyang-si, Republic of Korea) to determine group and serotype. In cases requiring confirmatory testing for nationally notifiable infectious diseases, sub-cultured *Salmonella* isolates were further characterized for *Salmonella* Typhi identification at the Seoul Institute of Health and Environment.

### 2.4. Statistical Analysis

Patients were classified into two groups based on their FA-GIP results: the FA-GIP-negative (FA-GIP−) group and the FA-GIP-positive (FA-GIP+) group. All measured variables and derived parameters were summarized using descriptive statistics and presented in tabular format. Categorical variables were compared between the two groups using the chi-square test. For continuous variables, statistical significance was assessed using an independent two-sample *t*-test. All tests were two-tailed, and a *p*-value ≤ 0.05 was considered statistically significant. Statistical analyses were performed using IBM SPSS Statistics 27 for Windows (IBM Corp., Armonk, NY, USA) and GraphPad Prism 8 (GraphPad Software, San Diego, CA, USA). Data were reported as mean ± standard deviation (SD) or as median and range, as appropriate. Missing data were handled as missing without imputation. Missing data accounted for <5% of the total dataset and were addressed using listwise deletion, as the proportion and pattern of missingness were unlikely to bias the primary analyses. Sensitivity checks confirmed that excluding cases with missing values did not meaningfully alter the overall results.

## 3. Results

### 3.1. Demographic Characteristics

Table 1 summarizes the demographic characteristics of the 161 retrospectively analyzed specimens. The majority of specimens (*n* = 143, 88.8%) were collected from hospitalized patients, whereas 9.3% and 1.9% of the samples were obtained from emergency department and outpatient cases, respectively. Based on the conventional age classification, 70 specimens (43.5%) were from adults (>21 years), whereas 91 specimens (56.5%) were from pediatric patients (≤21 years), indicating a higher proportion of pediatric patients in the study population. Additionally, slightly more samples were collected from female patients (*n* = 82, 50.9%) than from male patients (*n* = 79, 49.1%).

### 3.2. Distribution of Detected Pathogens in FA-GIP

**Figure 1:**
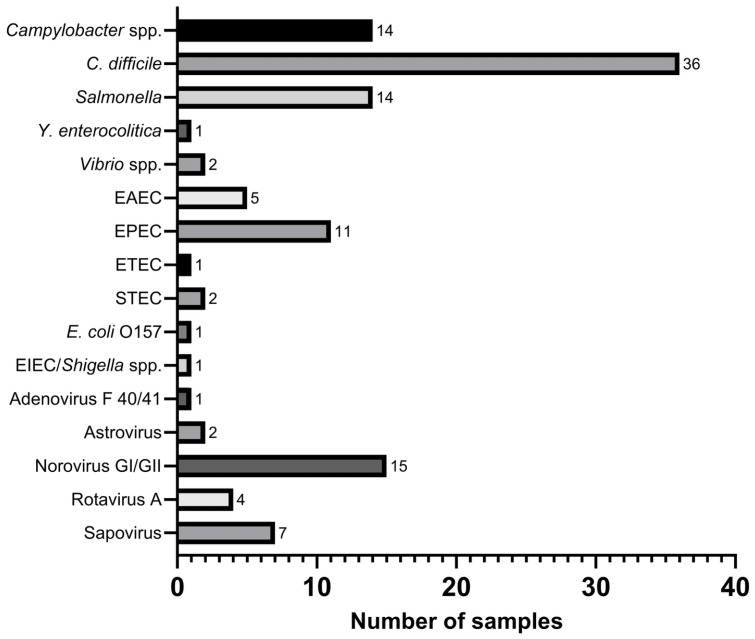
Distribution of causative pathogens in GIP+ samples. Bar chart showing the frequency of bacterial and viral pathogens detected in positive cases using the BioFire^®^ FA-GIP, including pathogens detected in mixed infections.

Figure 1 presents the distribution of positive pathogens detected using the FA-GIP assay. Of the 161 patients analyzed, 86 (53.4%) tested positive (FA-GIP+), whereas 75 (46.6%) tested negative (FA-GIP−), with a greater number of specimens collected from the FA-GIP+ group. A total of 117 pathogens were detected in the 86 FA-GIP+ samples. Of these 117 detected pathogens, 88 (75.2%) were of bacterial origin. The most frequently identified bacterial pathogen was *C. difficile* (40.9%), followed by *Campylobacter* spp. (15.9%) and *Salmonella* spp. (15.9%). The remaining 29 (24.8%) detected pathogens were viral in origin, with Norovirus GI/GII (51.7%) and Sapovirus (24.1%) being most prevalent. No parasitic infections were identified in this study cohort.

### 3.3. Coinfections

Table 2 presents the occurrence of coinfections among the detected pathogens. Among the 86 FA-GIP+ cases, 24 patients (27.9%) exhibited multiple pathogen infections. Specifically, dual-pathogen coinfections were identifiable in 18 cases (20.9%), triple-pathogen coinfections in 5 cases (5.8%), and quadruple-pathogen coinfections in 1 case (1.2%).

### 3.4. Overview of FA-GIP Detection Results

Table 3 presents the distribution of detected pathogens by age group. Among the 86 FA-GIP+ samples, the maximum number of potential pathogens detected per sample is four, with *C. difficile*, EAEC, STEC, and *E. coli* O157 being the most frequently co-detected pathogens.

Coinfections are observed in several cases. *C. difficile* is the most frequently detected pathogen in coinfections (*n* = 16), followed by EPEC, *Salmonella* spp., Norovirus GI/GII, and EAEC. Notably, coinfection is most commonly associated with EPEC (90.9%), whereas *Campylobacter* spp. exhibits the lowest coinfection rate, being detected in only 13 cases (14.3%).

Age-stratified analysis reveals that the ≥65 years age group accounts for the largest proportion of cases (*n* = 37, 31.6%). Within this group, the most prevalent pathogen is *C. difficile* (*n* = 13, 35.1%), followed by *Campylobacter* spp. and EPEC, which were both detected in four cases (10.8%). In pediatric patients, the 1–5 years age group has the highest prevalence of infections. The most frequently detected pathogen in this group is *C. difficile* spp. (*n* = 11, 34.4%), followed by Norovirus GI/GII (*n* = 8, 25.0%).

### 3.5. Turnaround Time

**Figure 2:**
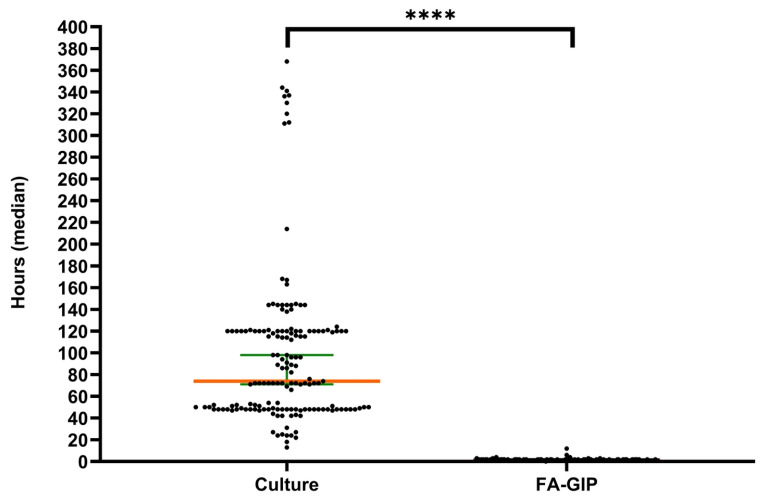
Turnaround time from specimen reception to result reporting. Scatter dot plot showing the turnaround time (TAT) median in hours for BioFire^®^ FA-GIP and stool culture (*n* = 161). Dots represent individual samples, and horizontal lines indicate the mean, with a 95% confidence interval. Statistical analysis was performed using GraphPad Prism 8 and applying the chi-square test. The **** indicates that the *p*-value < 0.0001.

Figure 2 presents a graphical comparison of the turnaround time (TAT) between stool culture and FA-GIP testing. The median TAT for FA-GIP is 1.4 h, whereas stool culture requires a median of 72.3 h (95% CI, 69.7–94.9%). FA-GIP testing yields results 70.9 h faster than those obtained using stool culture. The difference in TAT between FA-GIP and stool culture is statistically significant (*p* < 0.0001).

### 3.6. Comparison of FA-GIP and Stool Culture Performance

**Figure 3:**
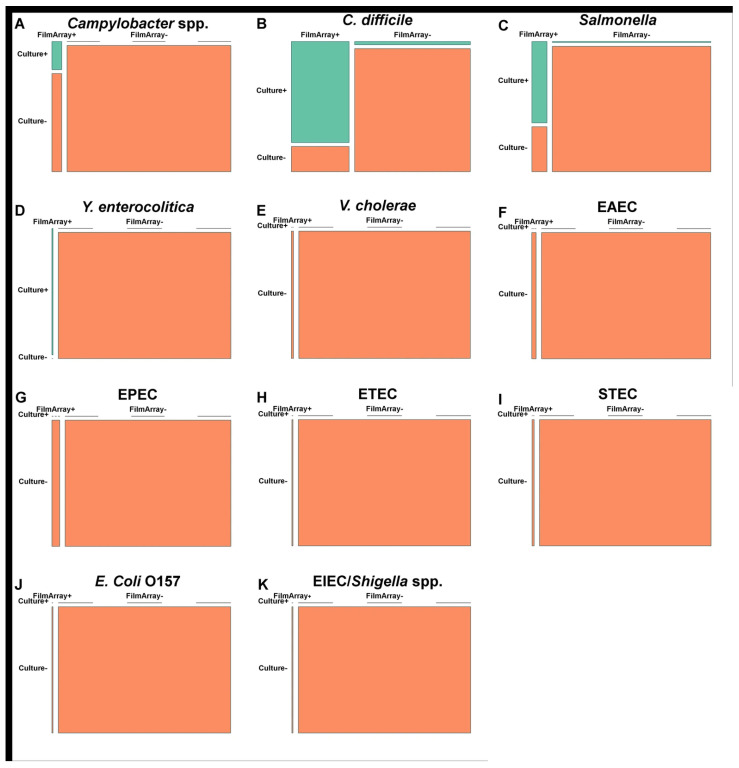
Performance summary of the BioFire^®^ FilmArray^®^ Gastrointestinal Panel (FA-GIP) versus conventional stool culture. Matched 2 × 2 comparison plots illustrate the diagnostic performance for 11 bacterial pathogens. Each subpanel (**A**–**K**) represents one pathogen. The green boxes represent true positives and true negatives, whereas the orange boxes represent false positives and false negatives. Exact case counts for the major pathogens are as follows: (**A**) *Campylobacter* spp.: true-positive (TP) = 2, false-positive (FP) = 0, false-negative (FN) = 7, and true-negative (TN) = 151; (**B**) *Clostridioides difficile*: TP = 16, FP = 1, FN = 4, and TN = 39; (**C**) *Salmonella* spp.: TP = 9, FP = 1, FN = 5, and TN = 146; (**D**) *Yersinia enterocolitica*: TP = 1, FP = 0, FN = 0, and TN = 160. For the remaining pathogens (panels **E**–**K**), prevalence is very low and performance cannot be meaningfully evaluated.

Figure 3 presents a performance comparison between stool culture and FA-GIP. The sensitivity and specificity of FA-GIP for *C. difficile* are 94.1% (95% CI, 56.3–94.3%) and 90.7% (95% CI, 89.1–99.9%), respectively. For *Campylobacter* spp., the sensitivity is 100% (95% CI, 28.1–60.0%) and the specificity is 95.6% (95% CI, 98.9–100.0%). *Salmonella* spp. exhibits a sensitivity of 90.0% (95% CI, 35.1–87.2%) and a specificity of 96.7% (95% CI, 97.2–100.0%). Due to the low prevalence of other pathogens, sensitivity calculations were not feasible; however, specificity remains above 95% for most pathogens.

### 3.7. Patient Characteristics and Clinical Outcomes

Table 4 summarizes the demographic and clinical characteristics of patients according to the FA-GIP results, along with the corresponding *p*-values for statistical comparisons. No significant differences are observed between the FA-GIP+ and FA-GIP− groups in terms of patients’ sex and age. However, statistically significant differences are identifiable between the two groups in the length of hospital stay, time to discharge based on FA-GIP results, duration of antibiotic use, modification or discontinuation of antibiotics, and the number of cases where patients were discharged or had their antibiotics discontinued without an antibiotic prescription.

## 4. Discussion

In this study, FA-GIP detected bacterial pathogens in 75.2% of the IGE samples, with *C. difficile* as the most frequently identified pathogen (40.9%), followed by *Campylobacter* spp. and *Salmonella* spp. (both 15.9%). Similarly, a study conducted by Torres-Miranda et al. in Washington, DC, USA, reported *C. difficile* as the most common pathogen (71/129, 55.0%), followed by *Campylobacter* spp. (27/129, 20.9%), and *Salmonella* spp. (16/129, 12.4%) [14].

*C. difficile* infection (CDI) is a globally recognized cause of severe infectious colitis. However, research on the increasing incidence of CDI in Asia remains limited, emphasizing the need for further investigation [15]. A Korean study also demonstrated a rising trend in CDI incidence, from 1.7 cases per 1000 patients in 2004 to 2.7 cases per 1000 patients in 2008 [16]. This underscores the necessity for continued surveillance and investigation of CDI epidemiology in the region. According to the 2020 FoodNet report, *Campylobacter* had the highest overall incidence rate (14.4 cases per 100,000 population), followed by *Salmonella* (13.3 cases per 100,000 population) [17]. These findings align with our study, where *Campylobacter* spp. and *Salmonella* spp. were the second and third most frequently identified bacterial pathogens, respectively, highlighting their significant role in IGE.

Among the 86 FA-GIP+ cases, 27.9% exhibited multiple pathogen infections. The most frequently detected coinfection pathogen was *C. difficile* (16 cases, 44.4%), followed by EPEC, *Salmonella*, Norovirus GI/GII, and EAEC. The presence of *C. difficile* in many premature infants likely reflects asymptomatic colonization rather than active infection [18]. Similarly, for *Salmonella* and Norovirus, it is important to consider their potential for prolonged shedding even after symptom resolution [19,20]. This highlights the complexity of interpreting IGE test results, as some pathogens may be detected in asymptomatic carriers or individuals may shed organisms post-infection. Given that FA-GIP is unable to quantify bacterial loads, it cannot differentiate between active infection and colonization. Therefore, complementary molecular assays capable of quantification should be incorporated into diagnostic workflows to aid clinical interpretation.

Our study also found that EPEC was the most frequently involved pathogen in coinfections (90.9%). FA-GIP sometimes shows false-positive results, particularly for EPEC, among diarrheagenic *E. coli*. Notably, EPEC is experiencing a resurgence in both developing countries and industrialized nations, with recent reports confirming its presence in Korea [21]. When multiple pathogens are detected, FA-GIP results may pose interpretational challenges for clinicians, who may, based on clinical judgment, sometimes dismiss certain pathogens as irrelevant, necessitating further investigation. This underscores the importance of supplementary diagnostic tools such as stool culture and quantitative multiplex molecular assays. However, identifying the primary causative pathogen in cases of coinfection remains challenging, necessitating the careful interpretation of results. Nonetheless, the rapid identification of potential pathogens is crucial for implementing appropriate infection control measures and optimizing treatment strategies.

Age has emerged as another factor influencing pathogen prevalence. In our cohort, individuals aged ≥65 years constituted the largest proportion of cases (37/117, 31.6%), with *C. difficile* being the most prevalent pathogen (35.1%), followed by *Campylobacter* spp. and EPEC (both 10.8%). According to Jump et al., older adults (≥65 years) are more susceptible to CDI than younger adults, with those requiring frequent healthcare services experiencing the highest risk for morbidity and mortality [22].

Cybulski et al. highlighted that commercial molecular assays, such as FA-GIP, outperform conventional culture-based diagnostics in terms of analytical sensitivity and turnaround time (TAT) [23]. In our study, FA-GIP significantly reduced TAT compared with that of stool culture (*p* < 0.0001). This shortened TAT facilitated earlier decision-making regarding antibiotic treatment and patient isolation, contributing to positive clinical outcomes. Due to the study design, we could not clearly distinguish between the use of empirical and targeted antibiotic therapy. This made assessing the direct impact of targeted antibiotic treatment difficult. Additionally, stool culture results for certain *Salmonella* Typhi samples needed to be outsourced to the Seoul Metropolitan Government Research Institute of Public Health and Environment, potentially introducing bias toward longer TATs for conventional methods.

Several studies have compared FA-GIP performance with other multiplex PCR assays and traditional culture methods. However, our study did not include comparisons with immunoassays or other PCR techniques and focused on comparison with conventional culture methods. In the comparative evaluation with stool culture, sensitivity could not be calculated for most pathogens due to their low prevalence, whereas specificity generally remained above 95%. The sensitivities of *C. difficile* and *Salmonella* spp. were <95%, likely reflecting bacterial culture limitations, particularly after antibiotic administration. Furthermore, PCR-based diagnostics may yield positive results even in asymptomatic carriers. PCR assays generally outperform stool culture in detecting symptomatic infections, as most patients undergoing IGE testing are symptomatic individuals seeking hospital care. Therefore, it is reasonable to assume that most *C. difficile*-positive cases detected by FA-GIP represent actual infections. The low sensitivity observed for other pathogens may be attributed to difficulties in bacterial culture or the stringent growth requirements for specific pathogens, resulting in discrepancies during culturing. Generally, seasonal variations in pathogen prevalence and the relatively low detection rates of certain pathogens during the study period posed challenges in sensitivity calculations.

The implementation of FA-GIP in clinical laboratories extends its implications beyond individual patient care to hospital resource utilization and infection control. Our study recorded significant reductions in hospital LOS, time to discharge, antibiotic use (duration, modification, and discontinuation), and the proportion of patients discharged without antibiotic prescriptions, after FA-GIP implementation. Beal et al. reported that replacing conventional culture methods with FA-GIP optimized patient isolation decisions and reduced nosocomial transmission [24]. The rapid turnaround of FA-GIP results can substantially contribute to cost reduction through shortened LOS, earlier discharge, and reduced isolation periods. Additionally, both positive and negative FA-GIP results hold clinical significance, influencing patient management decisions and optimizing infection control strategies and hospital resource utilization. Therefore, FA-GIP appears to provide significant value not only in terms of cost reduction but also in enhancing antibiotic stewardship practices.

Notably, a substantial proportion of patients experienced modifications or discontinuation of antibiotic therapy, highlighting the clinical impact of FA-GIP application on antibiotic usage. In our cohort, the rate of “antibiotics not prescribed or discontinued” was higher for the FA-GIP– group than that for the FA-GIP+ group. This pattern may be attributable to the higher likelihood of viral or nonbacterial etiologies in the FA-GIP– group, for which antibiotics are generally not indicated. Consequently, clinicians may have been more inclined to discontinue empirical antibiotics when FA-GIP results were negative, which could explain the observed difference between the two groups. Previous studies have demonstrated that the rapid turnaround of the FA-GIP results contributes to reducing antibiotic misuse while providing clinical benefits for hospitalized patients with acute diarrhea [25,26]. However, the role of multiplex PCR in antibiotic stewardship remains a subject of debate. One concern is that clinicians may reflexively initiate treatment based solely on FA-GIP results without confirmatory stool culture, potentially leading to unnecessary antibiotic prescriptions. Nevertheless, rapid pathogen identification via FA-GIP remains essential for formulating effective treatment plans and implementing appropriate infection control measures. These findings reinforce the clinical advantages of FA-GIP over conventional stool culture.

This study has several limitations. First, we performed a single-center retrospective analysis with a relatively small sample size, which may limit the generalizability of our findings. The relatively small sample size, particularly for low-prevalence pathogens, limited the statistical power of subgroup analyses and may have resulted in imprecise estimates of diagnostic performance. Furthermore, since the study was conducted at a tertiary medical center, the study population may have been skewed toward more severe cases. In addition, FA-GIP testing at our institution was typically ordered at the discretion of the attending physicians and may have been requested more frequently for inpatients, patients with severe or persistent symptoms, or those with underlying immunocompromising conditions. This possible selection bias could have contributed to the wide variation in length of hospital stay observed in the FA-GIP-negative group, as some patients may have been admitted for reasons other than acute gastroenteritis.

Moreover, etiological heterogeneity between the FA-GIP+ and FA-GIP– groups may have influenced the observed differences in clinical outcomes. Viral and bacterial infections differ in disease course and antibiotic requirements; therefore, some of the observed improvements may reflect these inherent differences rather than the direct effect of FA-GIP testing alone. Furthermore, the presence of coinfections made determining which pathogen represented the primary etiologic agent difficult, as the FA-GIP does not provide quantitative information. This limitation may have influenced the interpretation of clinical relevance for some detected organisms.

In this study, clinical decision-making regarding antibiotic initiation, modification, and discontinuation could not be fully evaluated. Thus, interpretations of antibiotic-related outcomes should be made with caution. Finally, the retrospective design inherently limited our ability to control for confounding variables and to confirm causal relationships between FA-GIP results and subsequent clinical decisions. Therefore, larger prospective studies are warranted to validate our findings and clarify the direct impact of FA-GIP testing on patient management and antibiotic stewardship.

In conclusion, this study shows that rapid results from FA-GIP were associated with the timely identification of causative pathogens in IGE, supporting more rapid clinical decision-making and patient management. These findings highlight the need for standardized interpretation guidelines to support consistent clinical decision-making when incorporating multiplex gastrointestinal panels into routine practice. Establishing clear criteria for interpreting positive, negative, and mixed results would help reduce variability among clinicians and improve the clinical utility of FA-GIP testing. Additionally, FA-GIP testing was linked to shorter turnaround times and differences in antimicrobial use between result groups. While these findings suggest potential benefits for antibiotic stewardship, decisions on patient isolation, hospitalization management, and infection control require careful clinical correlation. Taken together, the short turnaround time and broad pathogen detection spectrum indicate that FA-GIP is a valuable tool for IGE diagnosis in clinical laboratories.

## Figures and Tables

**Table 1:** Demographic characteristics of study specimens.

Patient Subset	Patient Sex and Age Data (Years) ^a^:												Total No. (%) of Specimens
	<1		1–5		6–12		13–21		22–64		≥65		
	M	F	M	F	M	F	M	F	M	F	M	F	
Emergency Room	0	0	0	1	1	0	0	1	2	3	3	4	15 (9.3)
Outpatient	0	0	0	1	2	0	0	0	0	0	0	0	3 (1.9)
Hospitalized	18	9	13	16	12	7	2	8	9	5	17	27	143 (88.8)
Total	18	9	13	18	15	7	2	9	11	8	20	31	161

^a^ M, Male; F, female.

**Table 2:** Coinfections detected in GIP+ specimens.

No. of Potential Pathogens in FilmArray GI Panel Result	No. of Specimens (*n* = 161)	% of Total (% Positives)
Detected (at least one)	86	53.4 (100)
One	62	38.5 (72.1)
Two	18	11.2 (20.9)
Three	5	3.1 (5.8)
Four	1	0.6 (1.2)

**Table 3:** Total number of FA-GIP+ by pathogen type and age group.

Potential Pathogen	Total No.	No. (% of Total) Associated with Coinfection	No. Detections in Age Group (Years):					
			<1 (*n* = 9)	1–5 (*n* = 32)	6–12 (*n* = 23)	13–21 (*n* = 9)	22–64 (*n* = 7)	≥65 (*n* = 37)
*Campylobacter* spp.	14	2 (14.3)	1	1	2	5	1	4
*C. difficile*	36	16 (44.4)	2	11	3	2	5	13
*P. shigelloides*	0	0(0)	0	0	0	0	0	0
*Salmonella*	14	6 (42.9)	0	5	4	1	1	3
*Y. enterocolitica*	1	0(0)	0	1	0	0	0	0
*Vibrio* spp.	2	2 (100)	0	0	0	0	0	2
*V. cholerae*	0	0(0)	0	0	0	0	0	0
EAEC	5	3 (60)	1	0	1	0	0	3
EPEC	11	10 (90.9)	0	3	3	1	0	4
ETEC	1	1 (100)	0	0	0	0	0	1
STEC	2	2 (100)	0	0	0	0	0	2
*E. coli* O157	1	1 (100)	0	0	0	0	0	1
EIEC/*Shigella* spp.	1	0(0)	0	0	0	0	0	1
Adenovirus F 40/41	1	1 (100)	0	1	0	0	0	0
Astrovirus	2	2 (100)	0	0	2	0	0	0
Norovirus GI/GII	15	6 (40)	3	8	3	0	0	1
Rotavirus A	4	1 (25)	2	0	0	0	0	2
Sapovirus	7	2 (28.6)	0	2	5	0	0	0
*Cryptosporidium*	0	0 (0)	0	0	0	0	0	0
*C. cayetanensis*	0	0 (0)	0	0	0	0	0	0
*E. histolytica*	0	0 (0)	0	0	0	0	0	0
*G. lamblia*	0	0 (0)	0	0	0	0	0	0
Total	117	55	9	32	23	9	7	37

**Table 4:** General demographic and clinical data of study participants.

Characteristics	FA-GIP− (*n* = 75) *n* (%)	FA-GIP+ (*n* = 86) *n* (%)	Total (*n* = 161) *n* (%)	*p*-Value *
Gender, *n* (%)				0.490
Female	36 (48.0)	46 (53.5)	82 (49.1)	
Male	39 (52.0)	40 (46.5)	79 (50.9)	
Age (years)				0.764
Mean (SD)	34.3 (35.7)	32.6 (34.8)	33.4 (35.1)	
Range	(0–91)	(0–98)	(0–98)	
Length of stay (Days)				<0.001
Mean (SD)	27.4 (32.7)	7.6 (9.0)	16.8 (25.2)	
Range	(1–174)	(1–53)	(1–174)	
Time to discharge from GIP results (Days)				<0.001
Mean (SD)	15.0 (19.1)	5.0 (6.5)	9.7 (14.7)	
Range	(0–95)	(0–40)	(0–95)	
Number of days on antibiotics (Days)				0.012
Mean (SD)	8.0 (11.0)	3.4 (7.9)	5.9 (9.9)	
Range	(0–30)	(0–30)	(0–30)	
Antibiotic treatment prior to test, *n* (%)				0.103
Yes	3 (5.1)	0 (0)	3 (2.7)	
No	56(94.9)	51(100)	107(97.3)	
Antibiotic treatment after test, *n* (%)				0.185
Yes	57 (96.6)	51 (100)	108 (98.2)	
No	2 (3.4)	0 (0)	2 (1.8)	
Antibiotic change and discontinuation, *n* (%)				0.011
Yes	24 (32.0)	13 (15.1)	37 (23.0)	
No	51 (68.0)	73 (84.9)	124 (77.0)	
Antibiotics not prescribed or discontinued, *n* (%)				0.008
Yes	16 (21.3)	35 (40.7)	51 (31.7)	
No	59 (78.7)	51 (59.3)	110 (68.3)	

* Welch’s *t*-test or Wilcoxon ranked-sum test (Mann–Whitney U-test) was used for continuous variables, and the chi-squared test was used for categorical variables. SD, standard deviation.

## Data Availability

The original contributions presented in this study are included in the article. Further inquiries can be directed to the corresponding author.
